# The cGAS-STING pathway in atherosclerosis

**DOI:** 10.3389/fcvm.2025.1550930

**Published:** 2025-04-25

**Authors:** Si-yu Wang, Yu-shan Chen, Bo-yuan Jin, Ahmad Bilal

**Affiliations:** ^1^ Heart Center/National Regional (Traditional Chinese Medicine) Cardiovascular Diagnosis and Treatment Center, The First Affiliated Hospital of Henan University of Traditional Chinese Medicine, Zhengzhou, Henan, China; ^2^ Department of Cardiology, The First Affiliated Hospital of Henan University of Traditional Chinese Medicine, Zhengzhou, Henan, China; ^3^ The First Clinical Medical College, Henan University of Traditional Chinese Medicine, Zhengzhou, Henan, China

**Keywords:** atherosclerosis, cGAS-STING pathway, risk factors, therapeutic potential, cGAS inhibitors, STING inhibitors

## Abstract

Atherosclerosis (AS), a chronic inflammatory disease, remains a leading contributor to cardiovascular morbidity and mortality. Recent studies highlight the critical role of the cGAS-STING pathway—a key innate immune signaling cascade—in driving AS progression. This pathway is activated by cytoplasmic DNA from damaged cells, thereby triggering inflammation and accelerating plaque formation. While risk factors such as aging, obesity, smoking, hypertension, and diabetes are known to exacerbate AS, emerging evidence suggests that these factors may also enhance cGAS-STING pathway, which amplifies inflammatory responses. Targeting this pathway offers a promising therapeutic strategy to reduce the burden of cardiovascular diseases (CVD). In this review, we summarize the mechanisms of the cGAS-STING pathway, explore its role in AS, and evaluate potential inhibitors as future therapeutic candidates. By integrating current knowledge, we aim to provide insights for developing novel treatments to mitigate AS and CVD burden.

## 1 Introduction

Cardiovascular disease (CVD) constitutes a major global health burden and remains the leading cause of mortality worldwide (1). Major adverse cardiac events (MACE) primarily contribute to CVD-related fatalities, and atherosclerosis (AS) is a predominant etiology for numerous cardiovascular disorders. AS is characterized by chronic inflammation, with lipid accumulation in the arterial wall, smooth muscle cell (SMC) proliferation, and fibrous matrix formation, which collectively culminate in the development of AS plaque (2). The retention of low-density lipoprotein cholesterol (LDL-C) induces local inflammation in the arterial endothelium. When this response is activated, it can precipitate plaque instability, plaque rupture, or thrombosis, thereby contributing to the onset of acute myocardial infarction (3). Inflammation is a pivotal factor throughout all stages of AS, acting as the foundation for physiological and pathological alterations during disease progression (4).

The cyclic GMP-AMP synthase (cGAS)-stimulator of interferon genes (STING) pathway is a pivotal part of the innate immune response, exerting a crucial influence on immune and inflammation reactions. The cGAS serves as an innate immune sensor of cytoplasmic double-stranded DNA (dsDNA). Under normal physiological conditions, DNA in mammalian cells is sequestered within the nucleus or mitochondria, and this makes cGAS in an inactive state. However, during pathogen invasion, the aberrant dsDNA with a phosphorylated backbone engages cGAS in a sequence-independent manner, triggering its activation. This activation leads to the synthesis of cyclic GMP-AMP (cGAMP) from ATP and GTP (5). As a second messenger, cGAMP triggers STING and the transcription factor type I interferon regulatory factor 3 (IRF3), thereby inducing robust innate immune responses (6). STING is a mediator of type I interferon (IFN-I) immune amboceptor. Compelling evidence from studies on chronic inflammation has established STING as a key inflammatory protein linked to chronic systemic inflammation (7–9). The endosymbiotic theory posits that mitochondria evolved from ancient bacteria, which share features such as circular genomes and hypomethylated CpG motifs (10, 11). Consequently, mitochondrial DNA (mtDNA) released into the cytoplasm or extracellular space is recognized by the immune system as damage-associated molecular patterns (DAMPs) through cGAS-mediated detection (12). Recent studies have demonstrated that under various pathological conditions, including oxidative stress, elevated pro-inflammatory cytokines, mitochondrial dysfunction, and viral infections, mtDNA is released and triggers the cGAS-STING pathway. The significance of the cGAS-STING pathway in AS has become increasingly clearer, with an abundance of foundational research providing a solid theoretical framework for advancing clinical investigations (13).

In this review, we briefly summarize the mechanisms of the cGAS-STING pathway while highlighting its pivotal role in AS. We underscore how the risk factors for AS affect the cGAS-STING pathway and explore molecular mechanisms demonstrating their influence on the pathway's activity. Furthermore, we evaluate the therapeutic potential of targeting this pathway in AS with a view to providing an effective therapeutic target for cardiovascular intervention. This analysis aims to provide a comprehensive resource for future research on AS and CVD, fostering the development of novel therapeutic strategies that can transform disease management paradigms.

## 2 Mechanism of the cGAS-STING pathway

### 2.1 Discovery and history of cGAS-STING signaling

**Figure 1:**
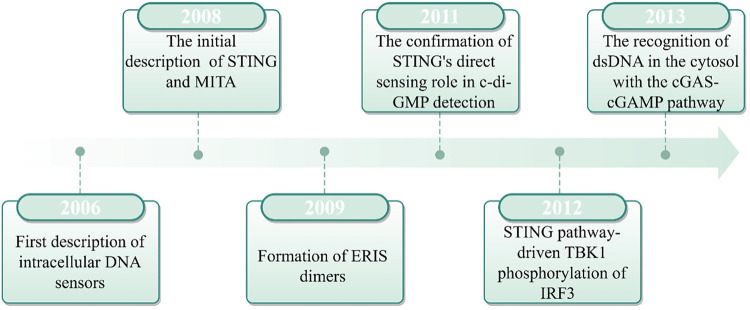
The history of cGAS-STING pathway.

In 2008, Hiroki Ishikawa and Glen N. Barber made a seminal breakthrough in antiviral immunity by identifying STING as a critical component of intracellular DNA-induced innate immune responses. Their research revealed that STING, which is localized in the endoplasmic reticulum (ER), functions upstream of TANK-binding kinase 1 (TBK1) and IRF3, thereby playing a critical role in the expression of IFNs (14). At the same time, independent research by Shu's team, John Cambier's team, and Jiang's team identified a protein complex (initially designated MITA, MPYS, and ERIS, respectively) that interact with TBK1 and IRF3 to induce IFN production. Subsequent studies confirmed these complexes represented the same protein, now collectively recognized as STING (15–17). STING was found to activate the nuclear factor kappa-light-chain-enhancer of activated B cells (NF-κB) and IRF3 transcriptional pathways in response to cytoplasmic DNA, thereby inducing IFN-α/β expression and mediating the innate immune response. However, the mechanism by which cytoplasmic DNA activates the STING pathway remained not fully understood. Until 2013, Chen's team discovered that upon pathogenic DNA infection, host cells are capable of producing cGAMP, thereby triggering the subsequent activation of STING (18). In another study, after affinity purification of cGAS, studies revealed that cGAS can directly recognize exogenous DNA and catalyze ATP/GTP conversion into cGAMP (19). The cGAMP, an intracellular messenger molecule produced by activated cGAS, activates STING, initiating the downstream immune response. This discovery established the cGAS-STING pathway as a cornerstone of innate immunity, which marked a major advance in our understanding of host defense mechanisms (Figure 1).

### 2.2 Summarize of cGAS-STING signaling

**Figure 2:**
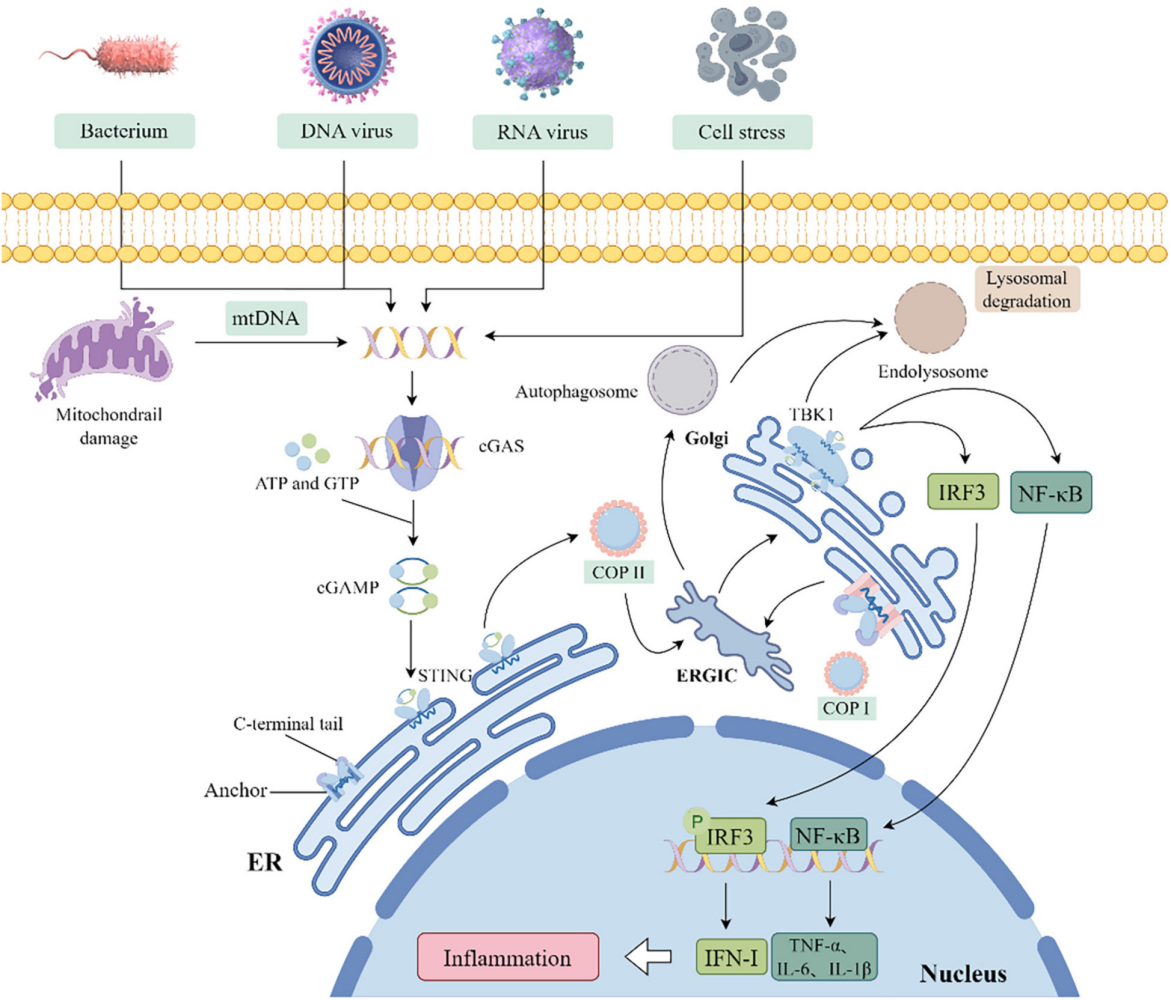
Schematic diagram of the cGAS-STING pathway activation and downstream signaling. (COP, coat protein; ERGIC, endoplasmic reticulum-Golgi intermediate compartment; ISGs, interferon-stimulated genes).

The cGAS-STING pathway, which evolved as a bacterial antiphage mechanism to detect exogenous and endogenous DNA, plays a pivotal role in inflammatory responses during infections, cellular stress, and tissue injury. It is intricately linked to diverse inflammatory, immune-related, and neoplastic disease states (Figure 2).

#### 2.2.1 Molecular structure of cGAS and STING

The cGAS is the initiator of the cGAS-STING pathway, composed of a nucleotide-binding domain (NBD) and a reverse transcriptase (RT) domain. The coordinated activity of these two domains endows cGAS with the ability to selectively recognize DNA, particularly in response to dsDNA breaks. This specific DNA binding event activates cGAS, thus enabling its catalytic activity to convert ATP and GTP into the second messenger cyclic GMP-AMP (2'3'-cGAMP) (20). STING, primarily localized in the ER membrane, serves as the receptor for cGAMP generated by cGAS. Its structure is characterized by an N-terminal extracellular domain, a transmembrane segment, and a C-terminal domain within the cell. Binding of cGAMP induces conformational changes in STING, facilitating its release from the ER membrane and promoting the formation of high molecular weight complexes. This activation initiates the downstream signal transduction cascade, leading to the production of IFN-I and inflammatory responses (21).

#### 2.2.2 Activation and effect mechanism of cGAS-STING

The cGAS-STING pathway, a critical component of the innate immune system, is activated by the dsDNA from viral, bacterial, or endogenous sources. The binding of cGAS to dsDNA allosterically activates its catalytic activity, thereby leading to the production of cGAMP and subsequent activation of STING proteins localized in the ER membrane (22). Dysregulation of the innate immune system's regulatory mechanisms can provoke various pathologically relevant aberrant immune reactions, impairing cellular and organismal homeostasis. Consequently, only dsDNA fragments exceeding a critical length and activation threshold trigger cGAS-dependent signaling, thereby preventing inappropriate activation by short dsDNA fragments (23–25). The binding of cGAS to dsDNA and subsequent cGAMP production are critical for triggering downstream signaling via STING. STING is essential for cell survival, proliferation, and induction of cell death, including necroptosis, apoptosis, and cellular senescence (26). In the absence of ligand, STING exists homodimeric and oligomerizes upon cGAMP binding. This process triggers STING translocation from the ER to the Golgi apparatus via COPII vesicles, where it recruits TBK1 and triggers its autophosphorylation. This event induces the expression of IRF1 and interferon-stimulated genes (ISGs), along with the production of pro-apoptotic genes, chemokines, and inflammatory mediators, thereby initiating downstream signaling (26–28). Moreover, the activation of STING triggers the activation of NF-κB and leads to the production of LC3, which initiates a non-canonical autophagy process that results in the formation of autophagosomes. Ultimately, STING within these autophagosomes, as well as STING molecules transported from the Golgi to lysosomes, is degraded in the lysosomes (28).

The latest research shows STING activation is not only contingent upon cGAMP binding but also crucially regulated by palmitoylation in its signaling cascade. Palmitoylation, a post-translational modification, covalently attaches palmitic acid to cysteine residue Cys91 of STING. This modification promotes STING oligomerization and its translocation from the ER to the Golgi apparatus (29, 30). Palmitoylation and STING oligomerization are essential for recruiting the TBK1-IRF3 complex and triggering downstream IFN-I production (31). Pharmacological inhibition of STING palmitoylation using small-molecule inhibitors such as H-151 in cell models abrogates STING activation, thereby reducing the expression of pro-inflammatory cytokines including IFN-β and IL-6 (32).

#### 2.2.3 cGAS-STING control of transcriptional responses

The cGAMP signaling pathway is critical for coordinating the synthesis of antiviral IFN-I and the induction of related gene products. This cascade is initiated when cGAMP-bound STING recruits TBK1, triggering TBK1 autophosphorylation. Activated TBK1 phosphorylates STING specifically at serine residue 366 (Ser366), thereby forming a stable STING-TBK1 complex. This complex subsequently recruits and phosphorylates IRF3, promoting IRF3 dimerization, nuclear translocation, and transcriptional activation of ISGs. In addition to regulating IFN-I and ISG expression, this activation also drives the transcription of inflammatory cytokines such as interleukin-6 (IL-6) and IL-12 (33–35). While IFN-I is critical for STING-mediated antiviral responses, it is not the sole pathway involved. STING also activates the NF-κB pathway via transcriptional regulation. Although TBK1 directly promotes NF-κB activation, this process is often complemented by IκB kinase *ε* (IKK*ε*), which acts upstream of the TGF-β-activated kinase 1 (TAK1) and IKK complex (36, 37). Furthermore, the cGAS-STING pathway can trigger non-canonical NF-κB signaling by inducing nuclear translocation of the p52/RELB complex. This signaling pathway is essential for regulating IFN-I and canonical NF-κB signaling, positioning these pathways as key modulators within the STING response network. Under specific conditions, cGAS-STING signaling may also intersect with the p53, signal transducer and activator of transcription 3 (STAT3), and p38 mitogen-activated protein kinase (p38 MAPK) pathways, thereby influencing cellular fate and alternative cytokine production (26, 37, 38).

### 2.3 mtDNA regulation of the cGAS-STING pathway

In addition to intracellular DNA, exogenous DNA, including cell-free DNA (cfDNA) and mtDNA, can activate the cGAS-STING pathway (39, 40). Upon entering the cytoplasm, exogenous DNA is recognized by cGAS, which binds to DNA and catalyzes cGAMP synthesis. cGAMP then activates STING, triggering downstream immune responses. Mitochondria, double-membraned semi-autonomous organelles unique to eukaryotes, are central to ATP generation, calcium homeostasis, apoptosis, reactive oxygen species (ROS) metabolism, and biosynthesis (41–45). Under physiological conditions, damaged mitochondria are selectively removed via mitophagy, a process critical for maintaining mitochondrial quality control (46). Under pathological conditions, mitochondrial damage can lead to mtDNA release into the cytoplasm, where it activates the cGAS-STING pathway similarly to exogenous DNA (47). Consequently, circulating mtDNA levels and mtDNA damage are significantly elevated in most CVD patients, showing a positive correlation with disease severity, chronic inflammation, and oxidative stress (48). Multiple studies show that mitochondria-released mtDNA activates the cGAS-STING pathway (49–51). This pathway specifically detects cytoplasmic self-DNA under pathological conditions, distinguishing this process from canonical antiviral responses that target foreign DNA (52). The cGAS-STING axis serves as a key sensor and downstream effector in mtDNA release-induced inflammatory responses (53), positioning mtDNA as the central activator of this signaling cascade. Under physiological conditions, cellular DNA is confined to the nucleus or mitochondria, and the cGAS-STING pathway is activated only upon sensing viral or bacterial DNA, inducing IFN-I response to protect neighboring cells from infection (54). However, during cellular aging, mitochondrial dysfunction leads to mtDNA release into the cytoplasm, triggering cGAS-STING-mediated IFN-I responses and inflammation. Enhanced mitophagy attenuates this process (50). Ouyang et al. reported that under stress, mtDNA is released into the cytoplasm via the mitochondrial permeability transition pore (mPTP), activating cGAS-STING and amplifying downstream inflammation (55). Genetic or pharmacological STING inhibition, combined with strategies to prevent mtDNA release, attenuates inflammation, whereas exogenous mtDNA delivery exacerbates mtDNA-induced inflammation by promoting the cGAS-STING pathway (55). Collectively, accumulating evidence suggests that the cGAS-STING pathway likely contributes to the pathogenesis of AS. Given the pathway's role in initiating inflammatory responses and the inflammatory microenvironment in AS, current data support a potential link between AS and cGAS-STING activation, although further studies are required to establish this association definitively.

## 3 The impact of the cGAS-STING pathway in AS

AS is a complex chronic inflammatory disease caused by multiple risk factors that collectively drive its initiation and progression. Specifically, LDL penetrates the arterial wall through dysfunctional endothelium, undergoes oxidation and modification, and accumulates within the vascular intima, thereby exacerbating endothelial dysfunction. During this process, altered leukocyte surface markers (e.g., monocytes and lymphocytes) combined with upregulated adhesion molecules facilitate leukocyte adhesion to the endothelium and subsequent trans-endothelial migration into the subendothelial space. Within this compartment, monocytes differentiate into macrophages while lymphocytes retain their phenotypes. These macrophages further differentiate into lipid-laden foam cells, forming early atherosclerotic lesions. Additionally, macrophages produce and secrete pro-inflammatory cytokines [e.g., IL-1, tumor necrosis factor-α (TNF-α)] and growth factors [e.g., platelet-derived growth factor [PDGF], fibroblast growth factor [FGF]]. These mediators play a critical role in promoting plaque progression and maintaining local inflammatory responses. Characterized by leukocyte infiltration, lipid accumulation, and foam cell formation, the atherosclerotic inflammatory microenvironment primarily affects medium-to-large arteries, leading to luminal stenosis and tissue ischemia. These pathological changes can culminate in severe clinical manifestations, with plaque rupture representing a life-threatening complication associated with high mortality (56). Although existing AS therapies (including pharmacological and interventional approaches) have improved patient outcomes, high morbidity and mortality rates persist. Emerging evidence underscores the cGAS-STING pathway as a key driver of AS progression, with activation observed in multiple cellular components of AS plaque, including vascular smooth muscle cells (VSMCs), endothelial cells, and immune cells (57–59).

### 3.1 Endothelial cells

Activation of the cGAS-STING pathway in endothelial cells upregulates adhesion molecules, including intercellular adhesion molecule-1 (ICAM-1) and vascular cell adhesion molecule-1 (VCAM-1), thereby promoting leukocyte adhesion and infiltration into the arterial wall. This is a critical step in early AS lesion formation (60). Furthermore, endothelial-to-mesenchymal transition (EndMT) accelerates endothelial dysfunction, promoting AS progression (61). Liu et al. demonstrated that mtDNA activates the cGAS-STING pathway during EndMT, indicating that mtDNA-triggered cGAS-STING signaling contributes to endothelial functional impairment (62). Additionally, studies have shown that mtDNA can activate autoimmune responses by evading DNA autophagy and degradation, accelerating AS lesion formation. This further supports the role for mtDNA in AS pathogenesis via the cGAS-STING pathway activation (63).

### 3.2 Macrophages

In macrophages, cGAS-STING pathway enhances scavenger receptor-mediated lipid uptake and foam cell formation. These lipid-laden macrophages contribute to AS plaque initiation and progression (64). Notably, animal studies demonstrate that in AS mice models, aortic lesions exhibit increased STING, cGAMP, and IFN-I expression, concomitant with lipid and macrophage accumulation. Conversely, suppressing STING expression can reduce AS lesions, lipid deposition, macrophage infiltration, and inflammatory cytokine levels (64). STING and cGAMP are also detected in human AS lesions. Huangfu et al. identified TDP43 as an upstream regulator in AS, activating the cGAS-STING pathway via mtDNA release and subsequent inflammatory responses (65). Fan et al. further revealed that activated gasdermin D (GSDMD) in macrophages promotes IL-1β release through pyroptosis and induces mitochondrial membrane permeabilization, leading to mtDNA liberation. This dual mechanism activates the cGAS-STING/NF-κB axis, augmenting IL-1β production and accelerating plaque formation (66). Additionally, IFN-I modulates macrophage phagocytic activity and skews T/B cell phenotypes, thereby exacerbating AS progression. Within plaque, cGAS-STING activation triggers IFN-I production, which weakens fibrous caps by promoting immune cell infiltration and matrix metalloproteinase (MMP) expression, enhancing plaque vulnerability (67).

### 3.3 VSMCs

In VSMCs, cGAS-STING pathway activation is associated with cell proliferation and phenotypic switching, collectively driving AS plaque progression (68). Attenuated collagen deposition by VSMCs results in thinner fibrous caps, increasing plaque instability. A recent study demonstrated that cGAS-STING activation, mediated by mitochondrial damage, exacerbates VSMCs phenotypic switching and premature senescence, leading to reduced collagen fiber production, thinner fibrous caps, and increased plaque fragility (69). Ge et al. further proposed that stromal interaction molecule 1 (STIM1), dissociated from STING during pathway activation, may promote AS pathogenesis. Specifically, STIM1 activation via extracellular Ca²^+^ influx or cGAS-STING pathway enhances endothelial inflammation, lipid accumulation, and VSMCs migration, thereby inducing pro-atherogenic VSMCs phenotypes and plaque instability (70). However, the precise mechanisms underlying STIM1/cGAS-STING signaling pathway in plaque expansion and vulnerability remain unclear. Additionally, emerging research has introduced the novel concept that AS is a SMC-driven tumor-like disease (71). This work highlights enhanced NF-κB activity in SMC-derived cells as a key mediator of phenotypic switching during AS progression. Nevertheless, the translational relevance of these findings to human AS requires further validation.

### 3.4 Oxidative stress

Activation of the cGAS-STING pathway induces oxidative stress within AS plaque, leading to reactive ROS production (60). Excessive ROS generation, combined with impaired antioxidant capacity, results in oxidative damage to the vascular wall. Accumulated ROS further promotes oxidative modification of cellular components, including lipid peroxidation, protein oxidation, DNA damage, and oxidation of LDL. These pathological processes amplify inflammatory responses, endothelial dysfunction, and tissue injury (72). Collectively, oxidative stress not only exacerbates inflammation and endothelial dysfunction but also accelerates lipid oxidation, driving AS plaque progression.

## 4 Effects of risk factors for as on the cGAS-STING pathway

**Figure 3:**
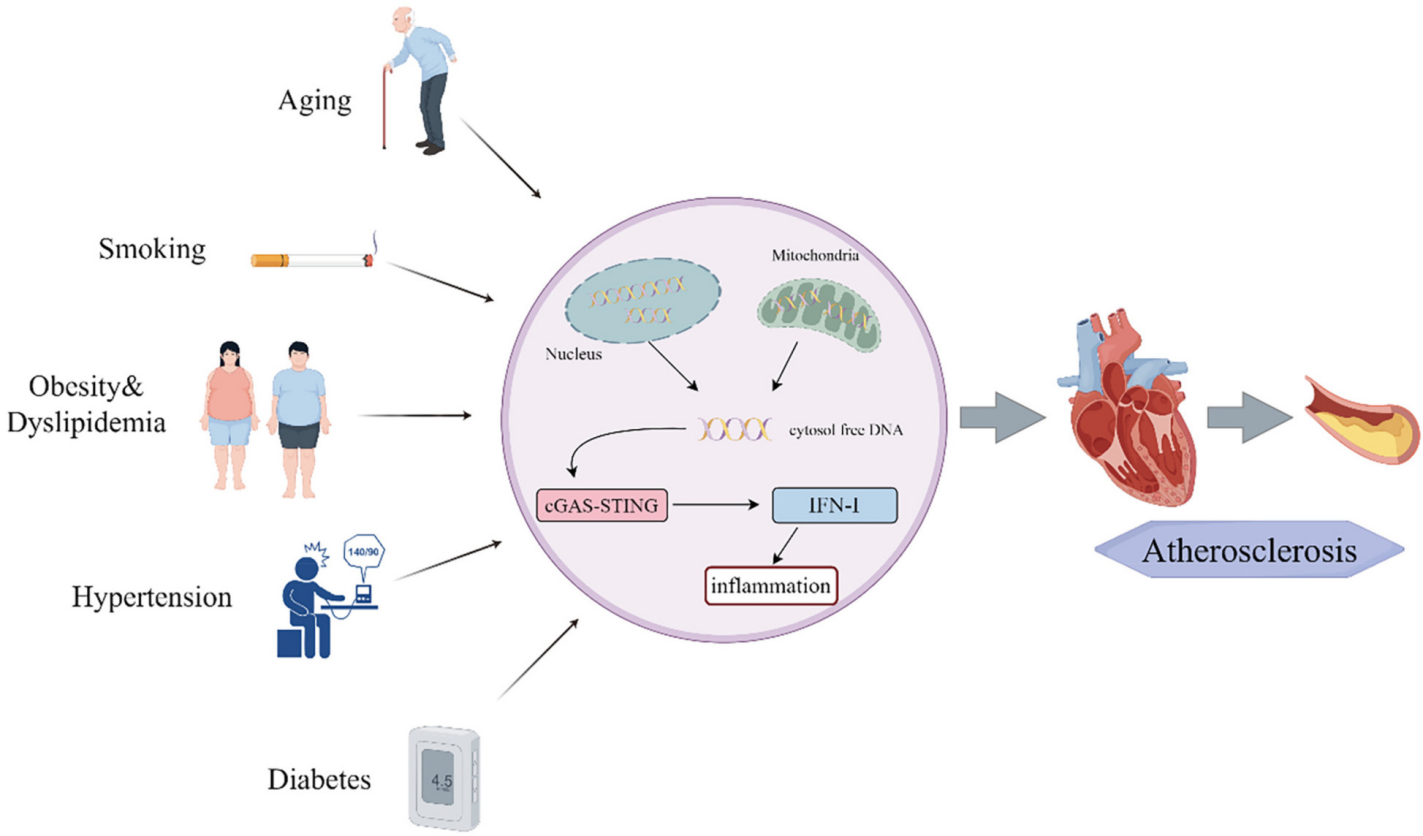
Risk factors of atherosclerosis influence the cGAS-STING pathway.

AS can lead to life-threatening complications, including angina pectoris and myocardial infarction, which pose significant threats to patient health. Recent studies have identified major AS risk factors—aging, smoking, obesity, hypertension, dyslipidemia, and diabetes (73–76) —which promote disease progression through activation of the cGAS-STING pathway. This mechanistic link provides a scientific rationale for implementing lifestyle modifications to mitigate AS progression (Figure 3).

### 4.1 Aging

AS predominantly affects individuals aged ≥40 years, with accelerated progression after 49 years of age. Recent epidemiological studies document a notable trend toward younger age at clinical onset (77). Estrogen exerts anti-atherosclerotic effects, which may explain the marked increase in AS incidence among postmenopausal women (78). Aging is a non-modifiable risk factor. As age advances, cardiovascular physiology experiences significant changes, such as arterial stiffening, endothelial dysfunction, and myocardial hypertrophy. Age-related systemic chronic inflammation plays a pivotal role in mediating these pathological changes (79). The senescence-associated secretory phenotype (SASP), a pro-inflammatory program in senescent cells, is regulated by the cGAS-STING pathway (80, 81). Takahashi et al. demonstrated that progressive downregulation of deoxyribonuclease 2 (DNase2) and three prime repair exonuclease 1 (TREX1) in senescent cells results in cytoplasmic DNA accumulation, activating the cGAS-STING pathway. This activation triggers SASP induction via IFN-β signaling (82). Notably, in aged murine myocardium and elderly human hearts, SASP is characterized by increased pro-inflammatory cytokines (e.g., IL-1β, IL-6, IL-8), which further promote mtDNA release and cGAS-STING activation, thereby sustaining SASP progression (83, 84). Moreover, the cGAS-STING pathway activation is critical for age-related endothelial dysfunction. Studies demonstrate that compared with younger individuals, aortic intima tissue from older adults exhibits markedly elevated levels of cGAS, STING, and phosphorylated IRF3 (p-IRF3) (85). These data suggest that aging-induced endothelial dysfunction occurs via the cGAS-STING pathway activation.

Furthermore, recent research highlights crosstalk between Yes-associated protein (YAP) transcriptional coactivator with a PDZ-binding domain (TAZ) and the cGAS-STING pathway as a key focus in age-related AS pathogenesis. Mechanistically, YAP/TAZ maintains nuclear integrity by directly modulating nuclear lamina proteins, thereby preventing nuclear DNA leakage into the cytoplasm and inhibiting cGAS-STING activation (86, 87). This regulatory function declines with aging, marked by reduced YAP/TAZ activity in multiple cell types and concomitant cGAS-STING hyperactivation (88). Aging-induced mitochondrial dysfunction exacerbates this imbalance by promoting mtDNA release into the cytosol, providing abundant ligands for cGAS. Concurrently, diminished YAP/TAZ-mediated suppression fails to counteract STING activation, leading to persistent pro-inflammatory cytokine production and establishment of a vicious cycle involving mtDNA release and inflammation (89). In the context of AS progression, age-related YAP/TAZ dysfunction indirectly disrupts lipid metabolism regulatory networks, promoting abnormal lipid deposition and subsequent intimal thickening and plaque formation (90). However, the precise molecular links between these pathways remain to be elucidated.

### 4.2 Smoking

Epidemiological evidence demonstrates a dose-dependent association between cigarette smoking and atherosclerotic progression, with smokers exhibiting a 2–6 fold increased risk of AS incidence and mortality compared to non-smokers (77, 91). Mechanistically, tobacco-derived compounds such as nicotine and polycyclic aromatic hydrocarbons promote cardiovascular pathogenesis through induction of chronic inflammation, endothelial dysfunction, and thrombotic activation (91). Ueda et al. have identified cfDNA as a critical mediator in smoking-induced AS progression. Among smokers, the serum of AS patients contains higher amounts of cfDNA, and there is an increased presence of cfDNA within AS lesions (40). Cigarette smoke extract (CSE) induces nuclear DNA damage and cytoplasmic accumulation in vascular cells, triggering the cGAS-STING pathway activation through recognition of fragmented nuclear DNA, which drives IL-6-mediated inflammatory responses (40). Concurrently, endothelial cell apoptosis induced by CSE leads to sustained cfDNA release, perpetuating a pro-inflammatory milieu that exacerbates plaque instability. Liu et al. found that side-stream smoke exposure (SSE), a second-hand smoke model, can induce mtDNA release, activating the cGAS-STING pathway (92). However, the full spectrum of factors triggering mtDNA damage and the associated mechanisms of mt-cfDNA release in AS remain incompletely understood. Additionally, emerging evidence highlights risks associated with e-cigarette use. Li et al. demonstrated that e-cigarettes increase circulating damaged mtDNA and upregulates Toll-like receptor 9 (TLR9) expression in macrophages, thereby augmenting pro-inflammatory cytokine production via both cGAS-STING and TLR9 signaling axes and accelerating AS development (93).

### 4.3 Obesity

Obesity represents a major risk factor for AS, characterized by hypertriglyceridemia, hypercholesterolemia, and frequent comorbidities with hypertension or diabetes. Recent studies highlight that insulin resistance (IR), prevalent in obese individuals, significantly elevates AS risk. Adipose tissue accumulation in overweight states, combined with inflammatory responses, promotes oxidative stress and mitochondrial damage, leading to mtDNA release into the cytoplasm. This mtDNA triggers cGAS-STING activation in adipose tissue and macrophages during obesity, thereby exacerbating chronic sterile inflammation. Systemic STING deficiency prevents high-fat diet (HFD)-induced adipose tissue inflammation, IR, and glucose intolerance (94, 95). Obesity promotes endothelial inflammation and induces pro-inflammatory responses in M1 macrophages. Mao et al. demonstrated that palmitic acid (PA)-induced mitochondrial damage and mtDNA release activate the cGAS-STING pathway and IFN production in both vitro and vivo models (96). This effect was attenuated by siRNA-mediated STING silencing or inhibition of IRF3 phosphorylation. In HFD-fed mice, IRF3 expression was markedly upregulated in adipose tissue and aortic walls. Conversely, STING-deficient (STING*^gt/gt^*) mice exhibited reduced IRF3 activation and were protected from HFD-induced vascular inflammation, IR, and glucose intolerance (96). Gong et al. found that dual knockout of v-akt murine thymoma viral oncogene homolog 2 (Akt2) and 5'-AMP-activated protein kinase (AMPK) activates the cGAS-STING pathway, exacerbating HFD-induced cardiac dysfunction characterized by oxidative stress, cardiomyocyte apoptosis, and pro-inflammatory cytokine release (97). Furthermore, Cruz et al. further reported that obesity promotes fatty acid-induced mtDNA translocation into the cytoplasm. In HFD-fed mice, global knockout of TBK1 or IRF3 ameliorated IR and reduced adipose tissue inflammation (98).

### 4.4 Dyslipidemia

AS is significantly influenced by dyslipidemia, which represents a major risk factor for the disease. Elevated levels of total cholesterol (TC), triglycerides (TG), very-low-density lipoprotein cholesterol (VLDL-C), lipoprotein(a) [Lp(a)], and apolipoprotein B (apoB), combined with reduced high-density lipoprotein cholesterol (HDL-C) and apolipoprotein A (apoA), are strongly associated with increased AS risk (99). Dyslipidemia frequently coexists with obesity, where lipid metabolic derangements contribute to AS pathogenesis and exacerbate cardiometabolic complications (100). Clinical evidence highlights a robust correlation between hypercholesterolemia and AS. Mechanistically, excessive lipotoxicity induces ROS overproduction, leading to mtDNA damage, the cGAS-STING pathway activation, and subsequent pyroptosis and hypertrophy in cardiomyocytes. Notably, AS patients exhibit significantly higher plasma mtDNA levels compared to healthy individuals (101). An et al. identified that IQ motif-containing GTPase-activating protein 1 (IQGAP1) drives ROS overproduction, thereby inducing the above mechanism (102). Their research further revealed that HFD-fed *Ldlr^−/−^* mice exhibited significantly elevated mtDNA expression compared to *ApoE^−/−^* mice. This mtDNA accumulation triggered cGAS-STING signaling, augmenting downstream phosphorylation of IRF3 and promoting the release of pro-inflammatory mediators [e.g., NOD-like receptor family pyrin domain containing 3 (NLRP3), IL-1β, IL-18] (102). Notably, while NLRP3 inflammasome activation was observed in this context, its precise mechanistic role in AS pathogenesis remains incompletely defined. Chen X. et al. identified oleoylethanolamide (OEA), an endogenous lipid homeostasis mediator, as a vasculoprotective agent that inhibits intimal calcification and prevents AS progression. Mechanistically, OEA suppresses hyperlipidemia-mediated ferritin deposition in rat VSMCs via the cGAS-STING pathway inhibition, thereby preventing vascular calcification (103).

### 4.5 Hypertension

Clinical and autopsy evidence demonstrate that hypertension independently elevates AS risk, with 60%–70% of coronary artery disease patients presenting comorbid hypertension and hypertensive individuals facing a 3–4-fold increased likelihood of developing coronary heart disease. The pathophysiological mechanisms involve hypertensive mechanical stress and pulsatile forces inducing endothelial dysfunction, characterized by increased intimal permeability to lipids, lipoprotein infiltration, monocyte adhesion, platelet aggregation, and VSMCs migration from the media to the intima. Endothelial injury and vascular remodeling represent cardinal pathological hallmarks of hypertension. The renin-angiotensin system (RAS) plays a central role in hypertension pathogenesis, while excessive sympathetic activation contributes to both hypertension and end-organ damage (104, 105). Recent studies demonstrate that hypertensive patients exhibit augmented circulating and urinary mtDNA, which directly impairs endothelial cell-mediated vasodilation by activating the cGAS-STING pathway. McCarthy CG et al. reported that male spontaneously hypertensive rats (SHR) display elevated plasma mtDNA levels concurrent with suppressed aortic mitophagy, evidenced by reduced Deoxyribonuclease II (DNase II) activity and autophagy-related proteins [Beclin-1, autophagy related 5 homolog [ATG5], microtubule-associated protein 1/2 light chain 3 [LC3-I/II]] (106). Guo X. et al. demonstrated that inducible nitric oxide synthase (iNOS) mediates cytosolic mtDNA accumulation and cGAS-STING activation under pressure overload conditions (107). Notably, preeclampsia (PE), a hypertensive disorder of pregnancy, may accelerate AS progression (108). Lv et al. noted that increased serum IL-1β in PE patients is associated with trophoblast-derived mtDNA activating NLRP3 inflammasomes, leading to endothelial cell damage characterized by overexpression of NLRP3, caspase-1 p20, IL-1β p17, and GSDMD, as well as impaired vasodilation (109). The cGAS-STING pathway contributes to PE pathogenesis through autophagy regulation: placentas from PE patients and rat models show upregulated cGAS, STING, and autophagy-related proteins. In the STING-overexpressing HTR-8/SVneo cell model, autophagy markers (P62 and LC3) are upregulated, whereas treatment with C176, a STING antagonist, reverses PE-like phenotypes *in vivo* (110).

### 4.6 Diabetes

Diabetes mellitus (DM) markedly elevates AS risk and exacerbates its progression compared to non-diabetic populations. Diabetic individuals often exhibit comorbid hyperlipidemia, with AS risk further compounded by coexisting hypertension. Additionally, diabetic patients frequently demonstrate elevated coagulation factor VIII levels and augmented platelet reactivity, accelerating thrombus formation in atherosclerotic arteries and contributing to luminal occlusion. Recent research highlights a strong association between IR and AS pathogenesis, with type 2 diabetic patients commonly presenting IR, hyperinsulinemia, and coronary artery disease (76). High expression of cGAS, STING, and TBK1 in adipocytes promotes IR and hyperglycemia (111). IRF3 exacerbates HFD-β-induced adipose tissue inflammation and IR, whereas IRF3 deficiency improves insulin sensitivity and glucose tolerance (112). Brahma-related gene 1 (BRG1) drives AS progression by promoting dsDNA accumulation, activating the cGAS-STING pathway, and exacerbating hyperglycemia-induced myocardial inflammation and apoptosis (113, 114). Yan et al. reported that the cGAS-STING pathway is activated in diabetic mouse hearts, characterized by increased TBK1 and IRF3 phosphorylation. STING knockdown via adeno-associated virus 9 (AAV9) attenuates pyroptosis, inflammatory responses, and diabetes-induced myocardial hypertrophy while preserving cardiac function (101). Additionally, Ma et al. further revealed that mtDNA accumulation activates the cGAS-STING pathway and downstream targets IRF3, NF-κB, IL-1l, and IL-1β during diabetic cardiomyopathy (DCM) (115).

### 5 Treatment of the cGAS-STING pathway in as

As highlighted in preceding sections, the cGAS-STING signaling pathway represents a promising therapeutic target for AS. This pathway, originally identified for its role in antiviral immunity, has emerged as a critical mediator of sterile inflammation in AS and other immune-mediated diseases. Current preclinical studies have evaluated small molecule inhibitors of cGAS-STING signaling, such as RU.521, PF-06928215, and C-176, which primarily block STING activation or downstream IRF3 phosphorylation, thus attenuating pro-inflammatory cytokine production. While these inhibitors show efficacy in experimental models, their clinical applications remain to be validated. However, several small molecule agonists targeting this pathway are undergoing clinical trials (e.g., NCT03172936, NCT04144140), providing mechanistic proof-of-concept for pathway modulation (116). In this section, we evaluate the therapeutic potential of cGAS-STING inhibitors in AS, analyzing their expected benefits and potential pitfalls. Targeting this pathway may offer novel strategies to mitigate AS progression and plaque formation (Tables 1, 2).

**Table 1:** cGAS inhibitors.

Medicine	Structure	Pharmacological mechanisms	Models	Reference
Aspirin	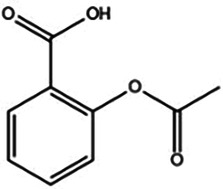	Maintains the acetylated inactive state of cGAS on Lys and avoids attack by dsDNA	Mice: *IRF3^−/−^*mice; Cell: 3T3-L1 preadipocytes and 3T3-F442A preadipocytes	(119)
Antimalarial drugs	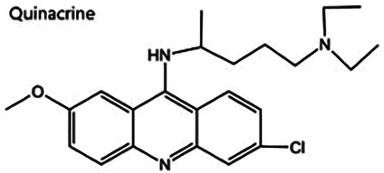 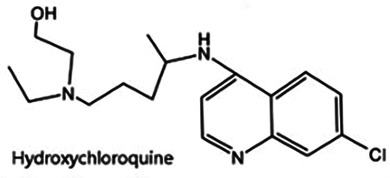 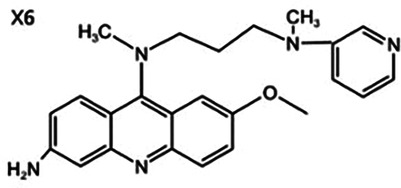	Blocking the binding of dsDNA to cGAS	Mice: *Trex1^−/−^ mice;* Cell: THP1 cell, PBMC	(121–123)
RU.521	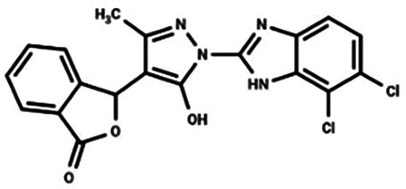	Inhibits the catalytic activity of ATP and GTP sites on cGAS, thereby affecting cGAMP production	Mice: *ApoE^−/−^* mice; Cell: HUVECs	(124)
PF-06928215	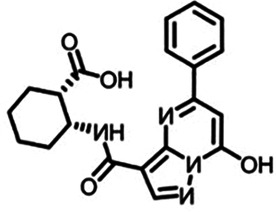	Interacts with cGAS at Lys362 and Lys350, thereby inhibiting the catalytic activity of cGAS by ATP and GTP, disrupting the production of cGAMP	Mice: Akt2-AMPK double knockout (DKO) mice	(125)
Suramin	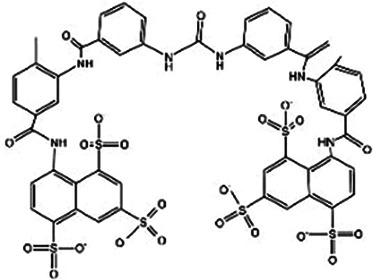	Competes with dsDNA for binding to cGAS, thereby preventing the interaction between DNA and cGAS	Cell: THP1 cell	(126)

**Table 2:** STING inhibitors.

Medicine	Structure	Pharmacological mechanisms	Models	Reference
Nitrofuran derivatives	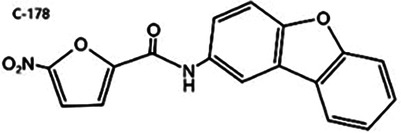 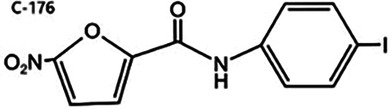	Binds to the Cys91 residue on the STING protein, inhibiting its palmitoylation	Mice: *Trex1^−/−^* mice; Cell: BMDMs, HEK293T cell	(26, 32)
H-151	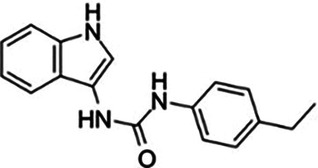	Selective covalent antagonist of STING, reduce phosphorylation of TBK1 and suppress palmitoylation of human STING	Mice: C57BL/6J mice; Cell: BMDMs	(32, 129)
NO_2_-OA	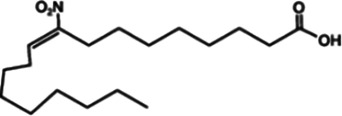	Targeting the palmitoylation site	Mice: Nos2*^−/−^* mice, STING-KO mice; Cell: RAW264.7, BMMs, THP-1	(132)
NO_2_-cLA	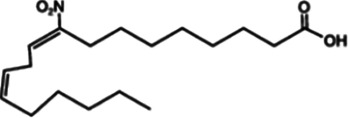	Targeting the palmitoylation site	Mice: Nos2*^−/−^* mice, STING-KO mice; Cell: RAW264.7, BMMs, THP-1	(132)
Astin C	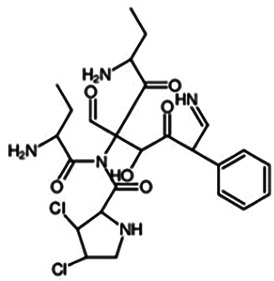	Binds to the C-terminal activation pocket of STING and reduce the recruitment of IRF3	Mice: *Trex1^−/−^* mice; Cell: RAW264.7, MEFs, hPBMCs, HEK cell	(134)
Compound 18	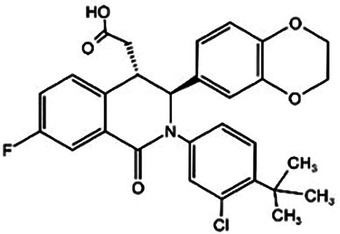	Inhibits STING by blocking the CDN binding domain	Cell: THP1 cell	(135)

### 5.1 cGAS inhibitors

Based on the molecular structure of cGAS and its interaction with DNA, the cGAS inhibitors mainly exert their effects through three mechanisms: First, mediating post-translational modifications of cGAS (e.g., aspirin inhibits cGAS activation via acetylation) (117); Second, blocking DNA binding to cGAS (e.g., antimalarial drugs competitively inhibit cGAS-DNA interaction) (57); Third, directly targeting the catalytic active site of cGAS (e.g., small-molecule inhibitors such as RU.521 and PF-06928215) (118). Subsequent sections will proceed to detail the key drugs associated with each mechanism.

#### 5.1.1 Aspirin

Aspirin, a non-steroidal anti-inflammatory drug (NSAID), is widely recognized for its ability to acetylate cyclooxygenase (COX) enzymes, inhibit platelet aggregation, and treat atherosclerotic cardiovascular disease. Dai et al. uncovered a novel mechanism by which aspirin suppresses the DNA-sensing cGAS-STING pathway. Specifically, aspirin directly targets cGAS protein and induces acetylation at critical lysine residues (Lys384, Lys394, and Lys414), thereby inhibiting cGAS enzymatic activity and IFN production. Notably, this acetylation does not interfere with cGAMP-induced phosphorylation of downstream factors IRF3 and TBK1 (119).

#### 5.1.2 Antimalarial drugs

Certain antimalarial drugs, such as hydroxychloroquine (HCQ), quinacrine (QC), and X6, exhibit therapeutic potential in inflammatory diseases and AS by inhibiting dsDNA binding to cGAS (120, 121). An et al. reported that HCQ, QC, and X6 selectively disrupt cGAS-dsDNA interactions, thereby suppressing IFN-β production. Their study further revealed that in *Trex1^−/−^* mice, both X6 and HCQ can inhibit the cGAS-STING pathway activation, reduce cGAMP expression, alleviate myocardial endocardial fibrosis, and mitigate inflammatory responses. Notably, X6 displayed enhanced therapeutic efficacy compared to HCQ (122). Wakiya et al. found that after administration of HCQ, the levels of TNF-α, IL-6, and IL-8 in serum were significantly reduced. This cytokine modulation contributes to improving the adipokine levels in patients and attenuating AS risk factors (123).

#### 5.1.3 Ru.521

Within the RU compound family, RU.521 has emerged as a cGAS enzymatic activity inhibitor that has garnered significant interest in cardiovascular research. This inhibitor exhibits minimal cytotoxicity at the IC_50_ concentration (≥50% cell viability retention) and competes with ATP**/**GTP for the active site of cGAS (124). Yu et al. indicated that intraperitoneal administration of RU.521 (5 mg/kg/day) to aged mice for 6 months downregulated cGAS expression in aortic tissues. This reduction may be attributed to the drug's long-term effects in mitigating inflammatory responses, preventing mitochondrial dysfunction, decreasing mtDNA release and ultimately inhibiting cGAS activation. Notably, these findings require further mechanistic validation (85). An et al. demonstrated that IQGAP1 can enhance mtDNA release, thereby activating the cGAS-STING pathway and promoting NLRP3-mediated pyroptosis. Strikingly, both RU.521 and C-176 were shown to reverse IQGAP1-induced NLRP3 pyroptosis in human umbilical vein endothelial cells (HUVECs) (102).

#### 5.1.4 PF-06928215

PF-06928215 is a substrate-competitive inhibitor that directly suppresses cGAS catalytic activity. Hall et al. revealed that PF-06928215 interacts with cGAS at Lys362 and Lys350, thereby inhibiting ATP**/**GTP binding and disrupting cGAMP production. This inhibitory effect is observed at concentrations as low as 200 nmmol/L (125). Gong et al. demonstrated that double knockout of Akt2 and AMPK exacerbates HFD-induced cGAS-STING pathway activation. PF-06928215 can inhibit cGAS activity (97). However, while these preclinical studies lack *in vivo* research support, they provide critical mechanistic insights and serve as foundational evidence for future translational research.

#### 5.1.5 Suramin

Suramin, a well-documented cGAS inhibitor, competes with dsDNA for binding to cGAS, thereby preventing the interaction between DNA and cGAS. This inhibitory mechanism reduces VSMCs migration and proliferation, consequently suppressing neointimal hyperplasia formation (126). These effects offer potential value for the treatment of CVD by alleviating inflammatory responses and fibrotic processes within the vasculature.

### 5.2 STING inhibitors

STING, a crucial component of the cGAS-STING pathway, can be targeted by inhibitors that interfere with its activation through the endogenous ligand cGAMP. These inhibitors primarily target the palmitoylation sites (e.g., C-176/178 and H-151) and the CDN binding sites (e.g., Astin C) (7, 127). The following is a concise summary of some representative STING inhibitors.

#### 5.2.1 Nitrofuran derivatives

Nitrofuran derivatives, including C-176, C-171, C-178, and C-170, have been recognized as cGAS pathway inhibitors through covalent modification of the Cys91 residue. This modification prevents palmitoylation-induced STING aggregation and impacts the IFN-1 responses (26). Among these, C-176 has been extensively investigated in CVD (32). Pham et al. demonstrated that the continuous intraperitoneal administration of C-176 (1 μmol, thrice weekly for 12 weeks) in AS models effectively inhibited macrophage activation and vascular inflammation. This intervention effectively attenuated the development of AS (64). Hua et al.'s *in vitro* study revealed that C-176 could impede PA-induced NLRP3-mediated pyroptosis and IFN-1 transcription in endothelial cells (128).

#### 5.2.2 H-151

H-151, a highly potent and selective covalent antagonist of STING, has been demonstrated to reduce TBK1 phosphorylation and suppress palmitoylation of human STING (32). While its role in AS remains understudied, emerging evidence highlights its therapeutic potential for other CVD. Hu et al. revealed that H-151 can inhibit the cGAS-STING-IRF3 pathway and inflammatory responses in cardiac macrophages during myocardial infarction (MI). This intervention reduces cardiac fibrosis and preserves cardiac function (129).

#### 5.2.3 Nitro fatty acids

Nitro fatty acids (NO_2_-FAs), including derivatives such as NO_2_-cLA and NO_2_-OA, are endogenous lipid mediators with well-documented cardioprotective properties (130, 131). These compounds inhibit the palmitoylation of STING through an S-nitro-alkylation reaction, thereby modulating the cGAS-STING pathway, which is integral to the immune response (132). NO_2_-OA has been shown to protect *ApoE*-deficient mice from AS plaque formation by reducing inflammatory cell infiltration and lipid deposition in aortic walls, suppressing pro-inflammatory cytokine (IL-1β, IL-6) production, and downregulating VCAM-1 expression (133).

#### 5.2.4 Astin C

Astin C, a natural cyclopeptide extracted from the traditional Chinese medicinal plant *Aster tataricus,* has been identified as a specific inhibitor of STING. Li et al. found that Astin C competitively occupies the C-terminal activation pocket of STING, thereby inhibiting the cGAS-STING pathway and reducing IRF3 recruitment. This molecular interaction effectively suppresses downstream inflammatory responses (134).

#### 5.2.5 Compound 18

Compound 18, recognized as the inaugural STING antagonist targeting the cyclic dinucleotide (CDN) binding domain, has been structurally characterized by x-ray crystallography to bind a single STING homodimer in an “open” conformation. This interaction disrupts cGAMP-mediated signaling cascades *in vitro*, thereby highlighting its potential as a modulator of STING activity (135).

### 5.3 Other mechanisms for inhibiting substance

Furthermore, various natural compounds modulate the cGAS-STING pathway through distinct mechanisms, potentially offering therapeutic or preventative benefits for AS. Tetrandrine (TET), a bisbenzylisoquinoline alkaloid extracted from Stephania tetrandra, exhibits anti-inflammatory properties. Li et al. revealed that TET inhibits the STING-TBK1/NF-κB signaling axis, thereby reducing inflammation in macrophages and attenuating AS lesion formation in HFD-fed *ApoE^−/−^* mice (136). These results position TET as a potential therapeutic candidate for AS. Another bioactive compound, aucubin—a bioactive compound with marked anti-inflammatory effects—has also been investigated. Liu et al. to inhibit mtDNA-induced activation of the STING/NF-κB pathway. This inhibitory effect effectively alleviated AS progression in HFD-*LDLr^−/−^*mice (137).

## 6 Conclusions and future prospects

Recently, there has been a significant surge in research interest and mechanistic insights into the cGAS-STING pathway. As previously mentioned, this cytosolic DNA sensing axis plays a pivotal role in initiating the innate immune response by detecting exogenous/endogenous DNA. Upon activation, the cGAS-STING pathway triggers downstream signaling cascades that culminate in the production of IFN-I and pro-inflammatory cytokines, thereby bridging innate and adaptive immunity to mount effective host defense mechanisms. However, when this pathway is disrupted, it leads to the occurrence of pathogen infections, inflammation, and even tumors.

In CVD, chronic inflammatory responses significantly affect the pathogenesis, progression, and clinical outcomes of these conditions, often resulting in unfavorable outcomes. Basic and clinical studies have indicated that the cGAS-STING pathway plays a crucial role in AS, offering novel therapeutic targets and strategies. The development and progression of AS are influenced by various factors, including aging, smoking, obesity, hypertension, and diabetes. These factors pose a severe threat to patients’ lives and health, causing tissue damage. During the progression of AS, the cGAS-STING pathway drives pathological progression through multifaceted mechanisms: Risk factors associated with AS act as triggers for the activation of the cGAS-STING pathway. Aging leads to cellular senescence and the release of mtDNA. Smoking induces DNA damage and the release of cfDNA. Obesity, hypertension, and diabetes promote mitochondrial dysfunction and oxidative stress. These factors collectively provide the initial impetus for the activation of the cGAS-STING pathway. Once activated, the pathway sets off a chain of pathological processes. In endothelial cells, the pathway upregulates adhesion molecules (e.g., ICAM-1, VCAM-1). This process promotes the adhesion of leukocytes to the endothelial surface and their subsequent infiltration into the arterial wall, which is a crucial step in the early formation of AS lesions. In macrophages, this pathway enhances lipid uptake and foam cell formation, key factors in plaque development. And it also stimulates the release of pro-inflammatory cytokines (e.g., IL-1β, TNF-α), further driving inflammation and plaque expansion. In VSMCs, the activation of the cGAS-STING pathway is associated with increased cell proliferation and phenotypic transformation. As a result, collagen secretion is reduced, the fibrous cap of the plaque becomes thinner, and the plaque instability increases. Moreover, the pathway also induces oxidative stress within plaque, generating ROS that exacerbate cellular damage, lipid peroxidation, and inflammation, collectively driving plaque progression. In summary, the cGAS-STING pathway integrates the effects of AS risk factors and orchestrates a series of cellular responses, including inflammatory reactions, endothelial dysfunction, lipid uptake in macrophages, changes in VSMCs, and oxidative stress. This pathway's central role in AS development and progression provides a solid basis for developing targeted therapeutic strategies to mitigate the disease.

A substantial body of research indicates that cGAS- and STING-targeting drugs hold enormous potential for improving AS. For example, aspirin, a cGAS antagonist, can directly target and induce acetylation at Lys384/394/414 of cGAS, thereby inhibiting its enzymatic activity and IFN production. This mechanism has been shown to effectively prevent and treat AS in preclinical models. Furthermore, the administration of the STING inhibitor C-176, a nitrofuran-based compound, demonstrated therapeutic efficacy in AS. Specifically, intraperitoneal injection of C-176 at a dose of 1 μmol thrice weekly for 12 weeks significantly attenuated macrophage activation and vascular inflammation in HFD-fed *ApoE^−/−^* mice, thereby reducing AS plaque burden. However, there are still unresolved issues with the cGAS-STING pathway. Firstly, the specific mechanisms regulating the activation and deactivation of the cGAS-STING pathway in different cellular contexts and stages of AS are not fully understood. While it's known that cytosolic dsDNA triggers the pathway, the precise factors determining the threshold and duration of activation, especially in response to endogenous DNA, remain unclear. Secondly, the cGAS-STING pathway intersects various metabolic and inflammatory signaling pathways in AS. The detailed crosstalk mechanisms between cGAS-STING and other pathways like NF-κB, NLRP3 inflammasome, and oxidative stress pathways, as well as the role of metabolic intermediates in modulating this crosstalk, remain to be explored. Moreover, although inhibitors targeting cGAS-STING show promise, their specificity and safety profiles in clinical settings require rigorous evaluation. The potential for off-target effects and the impact of long-term inhibition on immune function are critical concerns.

Therefore, in future research, we can delve into the specific mechanisms of the cGAS-STING signaling pathway in different stages and cell types of AS to provide a basis for precision medicine. It is undeniable that the cGAS-STING pathway is still in the preclinical stage. However, we believe that with a deeper understanding of its mechanisms and continuous optimization of treatment strategies, this pathway is expected to bring new breakthroughs in the treatment of AS, thereby reducing the incidence and mortality of cardiovascular diseases and providing better clinical treatments for patients.
