# Correction: The cGAS-STING pathway in atherosclerosis

**DOI:** 10.3389/fcvm.2025.1641100

**Published:** 2025-06-20

**Authors:** Si-yu Wang, Yu-shan Chen, Bo-yuan Jin, Ahmad Bilal

**Affiliations:** ^1^ Heart Center/National Regional (Traditional Chinese Medicine) Cardiovascular Diagnosis and Treatment Center, The First Affiliated Hospital of Henan University of Traditional Chinese Medicine, Zhengzhou, Henan, China; ^2^ Department of Cardiology, The First Affiliated Hospital of Henan University of Traditional Chinese Medicine, Zhengzhou, Henan, China; ^3^ The First Clinical Medical College, Henan University of Traditional Chinese Medicine, Zhengzhou, Henan, China

**Keywords:** atherosclerosis, cGAS-STING pathway, risk factors, therapeutic potential, cGAS inhibitors, STING inhibitors

Correction on The cGAS-STING pathway in atherosclerosis By Wang SY, Chen YS, Jin BY, Bilal A (2025). Front. Cardiovasc. Med. 12:1550930. doi: 10.3389/fcvm.2025.1550930

The affiliation order was erroneously presented in the published article. Affiliation “Heart Center/National Regional (Traditional Chinese Medicine) Cardiovascular Diagnosis and Treatment Center, The First Affiliated Hospital of Henan University of Traditional Chinese Medicine, Zhengzhou, Henan, China” was omitted from the paper. This affiliation has now been added for author(s) Si-yu Wang, Bo-yuan Jin and Ahmad Bilal.

The affiliation order has been corrected to

Si-yu Wang^1,2,3^, Yu-shan Chen^1,2*^, Bo-yuan Jin^1,2,3^ and Ahmad Bilal^1,2,3^

1 Heart Center/National Regional (Traditional Chinese Medicine) Cardiovascular Diagnosis and Treatment Center, The First Affiliated Hospital of Henan University of Traditional Chinese Medicine, Zhengzhou, Henan, China

2 Department of Cardiology, The First Affiliated Hospital of Henan University of Traditional Chinese Medicine, Zhengzhou, Henan, China

3 The First Clinical Medical College, Henan University of Traditional Chinese Medicine, Zhengzhou, Henan, China

The original version of this article has been updated.

